# Supplementary material for: Tree variability limits the detection of nutrient treatment effects on sap flux density in a northern hardwood forest
Source: PeerJ. 2022 Dec 12;10:e14410. doi: 10.7717/peerj.14410 (PMC9753739; doi:10.7717/peerj.14410)
Supplement: Table S1 — The response variable is sap flux density, with calcium silicate treatment, species, stand and “Early” vs “Late” as categorical fixed effects and various interactions. “Early” measurements were taken 2 or 3 years after the calcium silicate addition and “Late” were taken 4 or 6 years after the addition (Table 2). We did not report this model in the paper because the AIC was 44.4, compared to 1.48 for the model reported in Table 5. [file peerj-10-14410-s001.docx]

|  | **Df** | **Sum of Squares** | **F Value** | **Pr(>F)** |
| --- | --- | --- | --- | --- |
| “Early” vs “Late” | 1 | 0.028 | 1.39 | 0.24 |
| Stand | 4 | 0.58 | 1.00 | 0.41 |
| Treatment | 1 | 0.074 | 0.34 | 0.56 |
| Species | 4 | 0.20 | 0.70 | 0.59 |
| “Early” vs “Late” x Treatment | 1 | 0.0047 | 0.11 | 0.74 |
| Stand x Treatment | 4 | 0.16 | 0.55 | 0.70 |
| Stand x Species | 3 | 0.27 | 2.64 | 0.05 |
| Treatment x Species | 4 | 0.076 | 0.54 | 0.71 |
| Stand x Treatment x Species | 2 | 0.034 | 0.48 | 0.62 |
