# Supplementary material for: Tree variability limits the detection of nutrient treatment effects on sap flux density in a northern hardwood forest
Source: PeerJ. 2022 Dec 12;10:e14410. doi: 10.7717/peerj.14410 (PMC9753739; doi:10.7717/peerj.14410)
Supplement: Table S2 — The response variable is average daily sap flux density and the listed explanatory variables are categorical fixed effects two 2-way interactions. The three stands were studied in three different years (Table 3). We did not report this result in the paper because the AIC for this model was 97, compared to 86 for the one reported in Table 6. [file peerj-10-14410-s002.docx]

|  | Df | Sum of Squares | F Value | Pr(>F) |
| --- | --- | --- | --- | --- |
| Stand | 2 | 4.93 | 15.7 | <0.01 |
| N Treatment | 1 | 0.032 | 0.21 | 0.65 |
| P Treatment | 1 | 0.049 | 0.31 | 0.58 |
| Species | 2 | 0.17 | 0.54 | 0.59 |
| N x P Treatment | 1 | 0.15 | 0.93 | 0.34 |
| N Treatment x Stand | 2 | 0.028 | 0.09 | 0.92 |
| P Treatment x Stand | 2 | 0.22 | 0.69 | 0.50 |
| N Treatment x Species | 2 | 0.21 | 0.66 | 0.52 |
| P Treatment x Species | 2 | 0.18 | 0.57 | 0.57 |
| N x P Treatment x Stand | 2 | 0.49 | 1.56 | 0.22 |
| N x P Treatment x Species | 2 | 0.62 | 1.99 | 0.15 |
